# Factors associated with small- and large-for-gestational-age in socioeconomically vulnerable individuals in the 100 Million Brazilian Cohort

**DOI:** 10.1093/ajcn/nqab033

**Published:** 2021-04-07

**Authors:** Ila R Falcão, Rita de Cássia Ribeiro-Silva, Marcia Furquim de Almeida, Rosemeire L Fiaccone, Natanael J Silva, Enny S Paixao, Maria Yury Ichihara, Laura C Rodrigues, Mauricio L Barreto

**Affiliations:** The School of Nutrition, Federal University of Bahia, Salvador, Brazil; Centre for Data and Knowledge Integration for Health (CIDACS), Oswaldo Cruz Foundation, Salvador, Brazil; The School of Nutrition, Federal University of Bahia, Salvador, Brazil; Centre for Data and Knowledge Integration for Health (CIDACS), Oswaldo Cruz Foundation, Salvador, Brazil; School of Public Health, University of São Paulo, São Paulo, Brazil; Centre for Data and Knowledge Integration for Health (CIDACS), Oswaldo Cruz Foundation, Salvador, Brazil; Department of Statistics, Federal University of Bahia, Salvador, Brazil; Centre for Data and Knowledge Integration for Health (CIDACS), Oswaldo Cruz Foundation, Salvador, Brazil; Centre for Data and Knowledge Integration for Health (CIDACS), Oswaldo Cruz Foundation, Salvador, Brazil; Epidemiology and Population Health, London School of Hygiene and Tropical Medicine, London, United Kingdom; Centre for Data and Knowledge Integration for Health (CIDACS), Oswaldo Cruz Foundation, Salvador, Brazil; Institute of Collective Health, Federal University of Bahia, Salvador, Bahia, Brazil; Centre for Data and Knowledge Integration for Health (CIDACS), Oswaldo Cruz Foundation, Salvador, Brazil; Epidemiology and Population Health, London School of Hygiene and Tropical Medicine, London, United Kingdom; Centre for Data and Knowledge Integration for Health (CIDACS), Oswaldo Cruz Foundation, Salvador, Brazil; Institute of Collective Health, Federal University of Bahia, Salvador, Bahia, Brazil

**Keywords:** small-for-gestational age, large-for-gestational age, cohort, linkage, poor population

## Abstract

**Background:**

Evidence points to diverse risk factors associated with small- (SGA) and large-for-gestational-age (LGA) births. A more comprehensive understanding of these factors is imperative, especially in vulnerable populations.

**Objectives:**

To estimate the occurrence of and sociodemographic factors associated with SGA and LGA births in poor and extremely poor populations of Brazil.

**Methods:**

The study population consisted of women of reproductive age (14–49 y), whose last child was born between 2012 and 2015. INTERGROWTH 21st consortium criteria were used to classify weight for gestational age according to sex. Multinomial logistic regression modeling was performed to investigate associations of interest.

**Results:**

Of 5,521,517 live births analyzed, SGA and LGA corresponded to 7.8% and 17.1%, respectively. Multivariate analysis revealed greater odds of SGA in children born to women who self-reported as black (OR: 1.21; 95% CI: 1.19, 1.22), mixed-race (*parda*) (OR: 1.08; 95% CI: 1.07, 1.09), or indigenous (OR: 1.11; 95% CI: 1.06, 1.15), were unmarried (OR: 1.08; 95% CI: 1.07, 1.08), illiterate (OR: 1.47; 95% CI: 1.42, 1.52), did not receive prenatal care (OR: 1.57; 95% CI: 1.53, 1.60), or were aged 14–20 y (OR: 1.21; 95% CI: 1.20, 1.22) or 35–49 y (OR: 1.12; 95% CI: 1.10, 1.13). Considering LGA children, higher odds were found in infants born to women living in households with ≥3 inadequate housing conditions (OR: 1.11; 95% CI: 1.10, 1.12), in indigenous women (OR: 1.22; 95% CI: 1.19, 1.25), those who had 1–3 y of schooling (OR: 1.18; 95% CI: 1.17, 1.19), 1–3 prenatal visits (OR: 1.16; CI 95%: 1.14, 1.17), or were older (OR: 1.26; 95% CI: 1.25, 1.27).

**Conclusions:**

In poorer Brazilian populations, socioeconomic, racial, and maternal characteristics are consistently associated with the occurrence of SGA births, but remain less clearly linked to the occurrence of LGA births.

## Introduction

The newborn size is a product of the duration of pregnancy and rate of fetal growth. It is an important indicator of prenatal health and it has been associated with infant mortality, as well as short- and long-term morbidity (1). According to the Gaussian distribution of birth weight specific to sex, 3 main groups of live births have been conventionally defined: *1*) small-for-gestational-age (SGA: weight at gestational age <10th percentile); *2*) appropriate-for-gestational-age (weight at gestational age between the 10th and 90th percentiles); and *3*) large-for-gestational-age (LGA: weight at gestational age >90th percentile) (2–5).

In high-income countries, the prevalence of SGA and LGA is 4.6–15.3% and 5–20%, respectively (6, 7). Higher SGA burdens are evidenced in low- and middle-income countries (LMIC), as the prevalence of SGA varies from 7.0% in East Asia to 44.5% in South Asia (2). In Latin America and the Caribbean, 12.5% of all live births are considered SGA (8). The prevalence of macrosomia also varies among LMIC, ranging from 0.5% in India to 14.9% in Algeria (9). Variability in these estimates of SGA and LGA can be explained both by socioenvironmental factors and differences among populations, as well as by differences in the methodological approaches used to build these indicators (9–11).

There is evidence of a diversity of risk factors associated with SGA, such as: smoking, maternal short stature, underweight and low weight gain during pregnancy, chronic and infectious disease, nulliparity, extremes in maternal age, and placental pathology (12–15). The best-known risk factors for LGA are high pregestational BMI, pre-existing and gestational diabetes mellitus, the prior occurrence of LGA in pregnancy, and significant weight gain during pregnancy (12, 16–20). Some studies have shown SGA to be associated with social status, especially family income and schooling (21, 22); however, the role that socioeconomic factors play in LGA births is not well understood.

Despite significant improvements in maternal and child health indicators over recent decades in Brazil, neonatal and infant mortality rates remain unacceptably high, and regions with limited resources and specialized care (obstetric emergencies and high-quality prenatal care services) remain disproportionately affected (23, 24). Poverty and social inequality have been increasingly identified as main social causes underlying negative health outcomes in different populations (25, 26). Moreover, abnormal birth weight can introduce additional risks for both mothers and newborns living in poverty (27, 28).

A comprehensive understanding of the importance of socio-economic factors is imperative to develop strategies designed to improve maternal and infant health, especially in vulnerable populations in countries with high inequalities. In light of these considerations, our study aimed to estimate the frequencies of and identify socioeconomic factors associated with SGA and LGA in poor and extremely poor mothers in Brazil.

## Methods

### Population, study design, and data collection procedures

The present study considered baseline population-based data from the 100 Million (100M) Brazilian Cohort (29) linked with the National System of Information on Live Births (SINASC) from January 1, 2012 to December 31, 2015. The 100M Brazilian Cohort contains information on low-income families with a monthly per capita income <BRL200 (US$50). The cohort database consists of records containing socioeconomic data from 114,008,179 low-income individuals who applied for social assistance programs via the Unified Registry for Social Programs, and represents ∼55% of the entire Brazilian population (29).

Baseline data in the 100M Brazilian Cohort pertaining to women who gave birth between 2012 and 2015 were linked to the live birth registry from SINASC according 2 stages using Centre for Data and Knowledge Integration for Health-Record Linkage (30). The first was a deterministic linkage, and the second based on the similarity index. The novel record linkage tool considers the mother's name, mother's municipality of residence at time of registry/delivery, and mother's date of birth in the matching process. For current linkage, the estimated accuracy was >90% by year (0.94, 0.92, 0.91, and 0.93 for the years 2012, 2013, 2014, and 2015, respectively).

**FIGURE 1:**
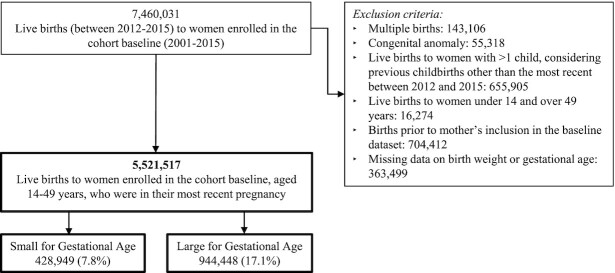
Study population.

The study population included the most recent live birth to women aged 14–49 y who were registered in the 100M Cohort at any time between 2001 and 2015 prior to giving birth (**Figure 1**). Only the most recent live birth to each woman was considered, because the inclusion of gestational age (measured in complete weeks) could influence the outcome of analysis. In an effort to avoid bias, multiple births, which accounted for <1.9% of the total live births in the presently studied population, as well as live births with congenital anomalies, were excluded, because these conditions are known to be strongly associated with low birth weight (31, 32) (Figure 1).

### Dependent variable

Newborn size was defined as appropriate for gestational age (between the 10th and 90th percentiles), SGA (<10th percentile), or LGA (>90th percentile), using sex-specific curves corresponding to singleton live births as established by the INTERGROWTH 21st Consortium (33) to classify weight at gestational age (24/0 to 42/0 gestational weeks). Gestational age was primarily measured according to the date of the mother's last menstrual period: the database contained 3,694,761 (62.8%) records with this information. Birth weight was recorded as the first live birth weight measurement in grams, and presents very high reliability as previously demonstrated by κ index values (34).

### Independent variables

The following covariates were included in the analysis. Variables related to maternal and newborn characteristics were obtained from SINASC records: maternal age range (14–19 y, 20–34 y, or 35–49 y), sex of newborn (male or female), and number of prenatal visits (≥7 visits, 4–6 visits, 1–3 visits, or none). Socioeconomic characteristics were obtained from the 100M Cohort database: marital status (married: married or in a stable relationship; unmarried: single, divorced, or widowed); mother's level of education (illiterate, 1–3 y, 4–7 y, or ≥8 y of schooling); self-declared race/skin color [white/Asian descent, mixed-race (*parda*), black, or indigenous]; housing conditions (adequate, 1–2 inadequacies, or 3+ inadequacies); and urban/rural area of residence. The variable referencing housing conditions is represented as the sum of the following circumstances: house construction material (adequate: brick/masonry; inadequate: wattle and daub, wood, or other), water supply (adequate: public system; inadequate: well/spring or other), lighting (adequate: home with electricity meter; inadequate: no meter), garbage collection (adequate: public collection service; inadequate: not collected), sanitary drainage (adequate: sewage system connection; inappropriate: sewage pit, ditch, or other), and family density (number of individuals in household ÷ number of rooms: ≤2 adequate; >2 inadequate). In each circumstance, a value of zero indicates an “adequate” housing condition, whereas 1 is considered “inadequate.” A maximum value of 6 would indicate that all housing conditions were inadequate, whereas zero would signify that all were adequate. This variable was categorized as no inadequacy, 1–2 inadequacies, or 3+ inadequacies.

### Statistical analysis

All the analysis conducted in this study was cross-sectional in nature. Socioeconomic, maternal, and live birth characteristics were summarized using frequency distributions. Collinearity was assessed for each independent variable by calculating changes in the estimation of the other covariates included in the model. Spearman rank correlation coefficients (ρ) were calculated for the quantitative variables birth order and maternal age. Multinomial (polytomous) logistic regression models were used to investigate factors associated with SGA and LGA. It is considered an appropriate technique when a response variable has >2 categories and does not assume a natural ordering. Birth weight according to gestational age was organized in 3 categories: appropriate-for-gestational-age (reference group), SGA, and LGA. Results are expressed as ORs with their respective 95% CIs.

**FIGURE 2:**
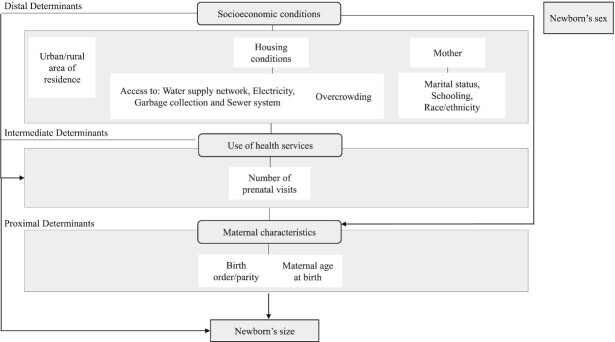
Analytical model detailing determinants of small- and large-for-gestational-age.

All analyses were conducted using the available covariates considered to be relevant and plausible in the literature (35–39). A conceptual hierarchy-based model was adopted for the introduction of variables (**Figure 2**). The initial model was adjusted for the following socioeconomic variables: education level, marital status, race/color, housing condition, urban/rural area of residence, in addition to the sex of the newborn and the year the mother entered into the cohort. In the second model, all variables contained in the previous model were maintained, with the inclusion of the number of prenatal visits. The final model included, in addition to the variables contained in the 2 previous models, the mother's age at the time of delivery. Given that inclusion in the cohort occurred dynamically, our analyses were also adjusted according to the year of the mother's entry in the cohort. Data were processed and analyzed using Stata version 15.1 (Stata Corp.) (40).

### Ethical considerations

The present research was approved by the institutional review board of the Collective Health Institute-Federal University of Bahia, and is a subproject under the umbrella of the main project entitled “Impacts of the Family Fund conditional cash-transfer program on mortality and hospitalization outcomes in Brazil” [*Impactos do Bolsa Família em desfechos de mortalidade e hospitalizações no Brasil* (in Portuguese)] (CAAE: 41,695,415.0.0000.5030).

## Results

A total of 5,521,517 live births were included in the study, of which 428,949 (7.8%) and 944,448 (17.1%) were classified as SGA and LGA, respectively (Figure 1).

**Table 1** lists the characteristics of the study population. Both SGA and LGA were slightly more common in rural areas, in households with ≥3 inadequacies, and in mothers who were indigenous or had fewer years of formal education. SGA was more frequent in babies born to single mothers compared with LGA.

**TABLE 1:** Small-for-gestational-age (SGA) and large-for-gestational-age (LGA) in full-term births from 2012 to 2015 according to variables related to mothers, live births, prenatal care, and socioeconomic conditions.

	*n* Missing		SGA^1^	LGA^1^
Variables	(%)	*n* (%)	*n* (%)	*n* (%)
Urban/rural area of residence	168,270 (4.1)			
Urban		3,957,206 (74.7)	303,196 (7.7)	665,888 (16.8)
Rural		1,342,669 (25.3)	109,154 (8.1)	241,787 (18.0)
Housing conditions	362,848 (8.7)			
No inadequacy		1,482,365 (29.4)	110,929 (7.5)	238,318 (16.1)
1–2 inadequacies		2,016,007 (40.0)	153,357 (7.6)	347,024 (17.2)
3+ inadequacies		1,539,243 (30.6)	128,001 (8.3)	274,714 (17.8)
Maternal race/ethnicity	315,086 (7.6)			
White/Asian descent		1,583,226 (31.0)	114,173 (7.2)	260,175 (16.4)
Mixed-race (*parda*)		3,075,059 (60.2)	244,098 (7.9)	537,974 (17.5)
Black		403,060 (7.9)	35,848 (8.9)	66,192 (16.4)
Indigenous		42,916 (0.8)	3763 (8.8)	9004 (21.0)
Marital status	48,775 (1.2)			
Married, civil union		2,909,389 (53.3)	212,615 (7.3)	521,053 (17.9)
Single, divorced, widowed		2,545,579 (46.7)	210,901 (8.3)	411,054 (16.1)
Maternal schooling	69,295 (1.7)			
≥8 y of study		3,704,186 (68.3)	270,873 (7.3)	614,328 (16.6)
4–7 y of study		1,438,325 (26.5)	123,758 (8.6)	254,257 (17.7)
1–3 y of study		243,154 (4.5)	22,149 (9.1)	49,651 (20.4)
Illiterate		41,209 (0.8)	4449 (10.8)	8584 (20.8)
Number of prenatal visits	23,982 (0.6)			
≥7 visits		3,351,441 (61.1)	242,741 (7.2)	558,962 (16.7)
4–6 visits		1,625,029 (29.6)	132,690 (8.2)	288,707 (17.8)
1–3 visits		413,571 (7.5)	38,510 (9.3)	76,740 (18.6)
None		99,412 (1.8)	11,567 (11.6)	15,398 (15.5)
Maternal age at birth	12 (0.0)			
20–35 y		3,780,015 (68.5)	273,117 (7.2)	670,804 (17.7)
14–20 y		1,253,184 (22.7)	117,136 (9.3)	168,508 (13.4)
35–49 y		488,301 (8.8)	38,694 (7.9)	105,133 (21.5)
Birth order	244,990 (5.9)			
2nd–4th child		2,891,438 (55.6)	188,274 (6.5)	563,712 (19.5)
≥5th child		343,156 (6.6)	27,476 (8.0)	78,149 (22.8)
1st child		1,964,808 (37.8)	182,939 (9.3)	255,722 (13.0)
Newborn sex	0 (0.0)			
Male		2,825,961 (51.2)	215,168 (7.6)	486,291 (17.2)
Female		2,695,556 (48.8)	213,781 (7.9)	458,157 (17.0)

1 SGA/LGA frequencies calculated by row. Adequate-for-gestational-age (AGA) has been omitted, but can be calculated from the information in Table 1. For example, %SGA in each category = (number of SGA individuals/number of individuals in the category) × 100.

**Table 2** illustrates the results of the multivariate analysis (final model). All steps up to this final model can be found in **Supplemental Table 1**. Due to collinearity between maternal age and parity/birth order (ρ = 0.56), the latter variable was removed from the adjusted model. The adjusted odds for SGA were higher in children born to women who self-reported as black (OR: 1.21; 95% CI: 1.19, 1.22), indigenous (OR: 1.11; 95% CI: 1.06, 1.15), or were unmarried (OR: 1.08; 95% CI: 1.07, 1.08). ORs were progressively higher with regard to fewer years of schooling (OR_illiterate_: 1.47; 95% CI: 1.42, 1.52), number of prenatal visits (OR_none_: 1.57; 95% CI: 1.53, 1.60), and for women in the upper and lower age ranges. Considering LGA children, higher odds were found in those born to women living in households with ≥3 inadequate housing conditions (OR: 1.11; 95% CI: 1.10, 1.12), in those who were indigenous (OR: 1.22; 95% CI: 1.19, 1.25), reported 1–3 y of schooling (OR: 1.18; 95% CI: 1.17, 1.19), attended 1–3 prenatal visits (OR: 1.16; 95% CI: 1.14, 1.17), or were older (OR: 1.26; 95% CI: 1.25, 1.27). The chance of LGA was not observed to progressively increase with fewer prenatal consultations (OR_none_: 0.95; 95% CI: 0.93, 0.97). In addition, younger women were found to have a much lower chance of giving birth to LGA newborns (OR: 0.72; 95% CI: 0.72, 0.73).

**TABLE 2:** Final model of the determinants of small-for-gestational-age (SGA) and large-for-gestational-age (LGA)^1^

	SGA	LGA
Variables	OR (95% CI)	OR (95% CI)
Urban/rural area of residence		
Urban	Ref	Ref
Rural	1.01 (1.00, 1.01)	1.02 (1.01, 1.02)
Housing conditions		
No inadequacy	Ref	Ref
1–2 inadequacies	1.01 (1.00, 1.01)	1.08 (1.08, 1.09)
3+ inadequacies	1.06 (1.05, 1.07)	1.11 (1.10, 1.12)
Maternal race/ethnicity		
White/Asian descent	Ref	Ref
Mixed-race (*parda*)	1.08 (1.07, 1.09)	1.07 (1.06, 1.07)
Black	1.21 (1.19, 1.22)	0.99 (0.98, 1.00)
Indigenous	1.11 (1.06, 1.15)	1.22 (1.19, 1.25)
Marital status		
Married, civil union	Ref	Ref
Single, divorced, widowed	1.08 (1.07, 1.08)	0.93 (0.92, 0.93)
Maternal schooling		
≥8 y of study	Ref	Ref
4–7 y of study	1.14 (1.13, 1.15)	1.09 (1.08, 1.09)
1–3 y of study	1.26 (1.24, 1.28)	1.18 (1.17, 1.19)
Illiterate	1.47 (1.42, 1.52)	1.14 (1.11, 1.18)
Number of prenatal visits		
≥7 visits	Ref	Ref
4–6 visits	1.11 (1.10, 1.12)	1.09 (1.09, 1.10)
1–3 visits	1.26 (1.24, 1.27)	1.16 (1.14, 1.17)
None	1.57 (1.53, 1.60)	0.95 (0.93, 0.97)
Maternal age at birth		
20–35 y	Ref	Ref
14–20 y	1.21 (1.20, 1.22)	0.72 (0.72, 0.73)
35–49 y	1.12 (1.10, 1.13)	1.26 (1.25, 1.27)
Newborn's sex		
Male	Ref	Ref
Female	1.04 (1.04, 1.05)	0.99 (0.98, 0.99)

1 Multinomial (polytomous) adjusted logistic regression was applied to all modeled variables and year of cohort entry. Ref, reference.

## Discussion

Our study aimed to identify frequencies and associated factors relative to SGA and LGA live births in poor and extremely poor women in Brazil. SGA occurrence was higher in mothers who were younger and older, mixed-race (*parda*), black or indigenous, single, divorced, or widowed, less educated, and who attended fewer prenatal visits. The occurrence of LGA was higher in households with inadequate housing conditions, in indigenous women, those who had less formal education, and in mothers who were older. In addition, LGA was less commonly observed in the absence of prenatal visits.

Our findings are consistent with data in the literature on disparities in birth outcomes due to socioeconomic conditions (8, 38, 39, 41–43), highlighting the influence of demographic, socioeconomic, and health service access factors on pregnancy outcomes. Our results indicate that the socioeconomic conditions of the households where the mothers lived were associated with LGA and SGA. Indeed, some of the poorest regions in Brazil are becoming progressively more impacted by emerging noncommunicable diseases, for example, obesity, diabetes, and hypertension, which increasingly affect women of reproductive age (44). In Brazil, the prevalence of overweight and obesity in adult women increased from 33.8% to 46.3% between 2008 and 2015 (44). Given this scenario, similar increases in LGA and fetal macrosomia are likely to occur (45–49). Similarly to the findings herein, previous studies have reported substantial disparities in the prevalence of SGA in women of different racial and ethnic backgrounds (50–52). Our results indicate that babies born to mixed-race (*parda*), black, or indigenous women were more likely to be SGA, and that mixed-race or indigenous women were more likely to give birth to LGA newborns compared with white mothers or those of Asian descent. Regarding the mechanisms of how maternal race impacts pregnancy-related outcomes, it is important to recognize how the experiences of racism and segregation can influence the outcome (53). This is especially true in cases where racism and residential segregation occur simultaneously, placing vulnerable women at a higher risk of facing a lack of investment and inferior conditions, which isolates them from amenities, opportunities, and resources, resulting in stressful conditions and/or the promotion of behaviors considered harmful to well-being, thus negatively affecting their reproductive health (46, 52–55). Other studies have demonstrated that unmarried (single) mothers are more likely to give birth to newborns with SGA than married mothers (56, 57). Being unmarried is increasingly recognized as a risk factor that can influence adverse results in perinatal health, potentially due to a lack of social support or increased stress (55, 57). However, the exact nature of such stressors remains unelucidated.

Our findings indicate that low levels of maternal education are associated with an increased chance of SGA/LGA in newborns. Education represents one of the most important dimensions of socioeconomic status in predicting the health of mothers and their children (58–60). Maternal education is a representative variable of social insertion in relation to access to material goods and information, and is an important factor in overcoming challenges to the health and social progress of vulnerable women (61). It is known that more highly educated women tend to seek out more information during pregnancy and solicit medical attention when appropriate (6, 62).

With regard to prenatal care, the chance of SGA at birth was observed to be higher in mothers with reduced numbers or no prenatal visits. The opposite was found with respect to LGA. A high number of prenatal visits might not necessarily be an indicator of high-quality prenatal care, and could be related to high-risk pregnancies requiring additional care (63). Accordingly, it is possible that women who had fewer prenatal consultations could have had fewer comorbidities, such as diabetes and obesity, and thus faced a reduced risk of having a baby with LGA. Another explanation could be due to the low occurrence of zero prenatal visits in LGA babies, which could lead to a spurious inverse association; however, this variable was found to be consistent in SGA. Because the SINASC database does not contain information related to comorbidities during pregnancy, we were not able to explore this hypothesis.

As we identified in our study, large-scale epidemiological studies also identified an increased risk of SGA in much younger and older mothers (64, 65). Advanced maternal age has been reported as a risk factor for LGA (37, 66, 67) whereas younger age has been associated with lower incidence of LGA (68).

Some of the variables associated with SGA and LGA are considered modifiable factors, and they could theoretically reduce the prevalence of these outcomes. Thus, socioeconomic and health interventions, such as financial support for the poorest pregnant women, equality policies, and access to education and high-quality health services, might improve maternal and child health to reduce the occurrence of SGA and LGA.

### Strengths and limitations

With respect to strengths, the use of a large dataset allowed the analysis of factors associated with growth curve deviations (SGA/LGA). Moreover, the concurrent analysis of SGA and LGA allowed the identification of common factors. Regarding limitations, it was not possible to investigate some classical biological risk factors associated with SGA or LGA, such as diabetes or obesity. The study was further limited by the fact that multiple methods of assessing gestational age could have led to measurement errors. However, a previous study evaluated the reliability of gestational age in the SINASC database and reported fair reliability (κ = 0.46) (34). Nonetheless, it is important to emphasize that the conclusions stated herein are not solely based on significant associations; the magnitude of effect size, as well as supporting data in the literature, further bolster the present findings. Importantly, the variables analyzed in this study were restricted to sociodemographic factors and the frequency of prenatal care visits. Because our results pertain to mothers living in poverty and extreme poverty, any attempts to generalize the present findings beyond the scope of the population considered herein must be made with due caution.

### Conclusion

In summary, the present large-scale retrospective study of adverse pregnancy outcomes (SGA and LGA) in poor and extremely poor Brazilian mothers identified associations with sociodemographic factors and prenatal care visits, which highlights the importance of monitoring birth weight in vulnerable populations. Although socioeconomic and maternal characteristics were observed to be consistently strongly associated with SGA births, it is necessary to further investigate relations between LGA and schooling and number of prenatal visits. We emphasize the importance of maintaining financial support for vulnerable mothers and social protections for pregnant workers, as well as the promotion and implementation of policies fostering equity among genders and races. Finally, it is essential to improve access to high-quality primary services, such as education and health, in addition to increasing efforts focused on preventing teenage pregnancy. We further recommend the undertaking of longitudinal studies designed to elucidate the long-term consequences of SGA and LGA.

## Supplementary Material

nqab033_Supplemental_File

## Data Availability

Data described in the manuscript, code book, and analytic code will be made available upon request.

## Notes

This study was funded by MCTI/CNPq/MS/SCTIE/Decit/Bill & Melinda Gates Foundation's Grandes Desafios Brasil—Desenvolvimento Saudável para Todas as Crianças (call number 47/2014). CIDACS and the 100 Million Cohort received core support from the Wellcome Trust (grant number 202912/Z/16/Z); Health Surveillance Secretariat, Ministry of Health, Brazil, Bahia State; Fundação de Amparo à Pesquisa do Estado da Bahia (FAPESB); Financiadora de Estudos e Projetos-FINEP, Secretariat of Science and Technology of the State of Bahia-SECTI. IRF received a doctoral scholarship from Fundação de Amparo à Pesquisa do Estado da Bahia (FAPESB) (grant number BOL2330 / 2016). ESP is a fellow supported by the Wellcome Trust (grant number 213589/Z/18/Z).

Supplemental Table 1 is available from the “Supplementary data” link in the online posting of the article and from the same link in the online table of contents at https://academic.oup.com/ajcn/.

Abbreviations used: CIDACS, Centre for Data and Knowledge Integration for Health; LGA, large-for-gestational-age; LMIC, low- and middle-income countries; SGA, small-for-gestational-age; SINASC, National System of Information on Live Births.
