# Supplementary material for: Factors associated with small- and large-for-gestational-age in socioeconomically vulnerable individuals in the 100 Million Brazilian Cohort
Source: Am J Clin Nutr. 2021 Apr 7;114(1):109–16. doi: 10.1093/ajcn/nqab033 (PMC8246620; doi:10.1093/ajcn/nqab033)
Supplement: nqab033_Supplemental_File [file nqab033_supplemental_file.docx]

**Factors associated with small- and large-for-gestational-age among socioeconomically vulnerable individuals in the 100 Million Brazilian Cohort**

Ila R. Falcão

Online Supplementary Material

| **Supplementary Table 1. Adjusted models* of the determinants of Small for Gestational Age (SGA) and Large for Gestational Age (LGA).** | | | | | | | |
| --- | --- | --- | --- | --- | --- | --- | --- |
| **Variables** | **SGA** | | |  | **LGA** | | |
|  | **OR (CI 95%)** | | |  | **OR (CI 95%)** | | |
|  | **Model (M) 1**  **(Distal variables)** | **Model 2 (M1+Intermediate variables)** | **Model 3 (M2+Proximal variables)** |  | **Model 1**  **(Distal variables)** | **Model 2 (M1+Intermediate variables)** | **Model 3 (M2+Proximal variables)** |
| **Newborn's sex** |  |  |  |  |  |  |  |
| Male | Ref | Ref | Ref |  | Ref | Ref | Ref |
| Female | 1.05 (1.04; 1.05) | 1.05 (1.04; 1.05) | 1.05 (1.04; 1.06) |  | 0.97 (0.97; 0.98) | 0.97 (0.97; 0.98) | 0.97 (0.97; 0.98) |
| **Urban/rural area of residence** |  |  |  |  |  |  |  |
| Urban | Ref | Ref | Ref |  | Ref | Ref | Ref |
| Rural | 0.99 (0.98; 1.00) | 1.00 (0.99; 1.01) | 1.00 (0.98; 1.01) |  | 1.02 (1.01; 1.03) | 1.02 (1.01; 1.03) | 1.03 (1.02; 1.04) |
| **Housing conditions** |  |  |  |  |  |  |  |
| No inadequacy | Ref | Ref | Ref |  | Ref | Ref | Ref |
| 1-2 inadequacies | 1.01 (1.00; 1.02) | 1.01 (1.00; 1.02) | 1.00 (0.99; 1.01) |  | 1.06 (1.05; 1.06) | 1.06 (1.05; 1.07) | 1.08 (1.07; 1.08) |
| 3+ inadequacies | 1.09 (1.08; 1.11) | 1.07 (1.06; 1.08) | 1.06 (1.04; 1.07) |  | 1.02 (1.01; 1.03) | 1.03 (1.02; 1.04) | 1.06 (1.05; 1.07) |
| **Maternal race/ethnicity** |  |  |  |  |  |  |  |
| White/Asian descent | Ref | Ref | Ref |  | Ref | Ref | Ref |
| Brown/Mixed *“parda”* | 1.10 (1.09; 1.11) | 1.08 (1.07; 1.09) | 1.08 (1.07; 1.09) |  | 1.03 (1.02; 1.03) | 1.03 (1.03; 1.04) | 1.04 (1.03; 1.05) |
| Black | 1.23 (1.21; 1.25) | 1.21 (1.19; 1.22) | 1.22 (1.20; 1.24) |  | 0.97 (0.96; 0.98) | 0.97 (0.96; 0.99) | 0.94 (0.93; 0.96) |
| Indigenous | 1.17 (1.12; 1.22) | 1.11 (1.06; 1.16) | 1.14 (1.09; 1.19) |  | 1.00 (0.97; 1.04) | 1.03 (0.99; 1.06) | 0.99 (0.96; 1.03) |
| **Marital status** |  |  |  |  |  |  |  |
| Married, civil union | Ref | Ref | Ref |  | Ref | Ref | Ref |
| Single, divorced, widow | 1.13 (1.13; 1.14) | 1.12 (1.11; 1.12) | 1.08 (1.07; 1.08) |  | 0.85 (0.85; 0.86) | 0.86 (0.86; 0.87) | 0.92 (0.92; 0.93) |
| **Maternal schooling** |  |  |  |  |  |  |  |
| ≥8years of study | Ref | Ref | Ref |  | Ref | Ref | Ref |
| 4 to 7 years of study | 1.20 (1.19; 1.21) | 1.17 (1.16; 1.18) | 1.24 (1.23; 1.25) |  | 1.01 (1.01; 1.02) | 1.02 (1.02; 1.03) | 0.95 (0.94; 0.95) |
| 1 to 3 years of study | 1.30 (1.28; 1.33) | 1.26 (1.24; 1.28) | 1.39 (1.37; 1.42) |  | 1.19 (1.18; 1.21) | 1.21 (1.19; 1.23) | 0.97 (0.95; 0.98) |
| Illiterate | 1.57 (1.51; 1.63) | 1.47 (1.41; 1.53) | 1.60 (1.53; 1.66) |  | 1.20 (1.16; 1.24) | 1.23 (1.19; 1.27) | 0.90 (0.87; 0.93) |
| **Number of prenatal visits** |  |  |  |  |  |  |  |
| 7 or more visits | - | Ref | Ref |  | - | Ref | Ref |
| 4 to 6 visits | - | 1.12 (1.12; 1.13) | 1.15 (1.14; 1.16) |  | - | 0.95 (0.94; 0.96) | 0.93 (0.93; 0.94) |
| 1 to 3 visits | - | 1.32 (1.30; 1.33) | 1.39 (1.37; 1.41) |  | - | 0.87 (0.86; 0.88) | 0.83 (0.82; 0.84) |
| None | - | 1.62 (1.58; 1.66) | 1.74 (1.69; 1.78) |  | - | 0.88 (0.86; 0.90) | 0.82 (0.80; 0.84) |
| **Maternal age at birth** |  |  |  |  |  |  |  |
| 20 to 35 years | - | - | Ref |  | - | - | Ref |
| 14 to 20 years | - | - | 1.04 (1.03; 1.05) |  | - | - | 0.76 (0.75; 0.77) |
| 35 to 49 years | - | - | 1.20 (1.18; 1.21) |  | - | - | 1.17 (1.16; 1.19) |
| **Birth order** |  |  |  |  |  |  |  |
| 2nd to 4th child | - | - | Ref |  | - | - | Ref |
| 5th or higher | - | - | 1.05 (1.04; 1.07) |  | - | - | 1.16 (1.14; 1.17) |
| 1st child | - | - | 1.48 (1.47; 1.50) |  | - | - | 0.66 (0.66; 0.67) |
| *Multinomial (polytomous) adjusted logistic regression was applied to all modeled variables and year of cohort entry. | | | | | | | |
